# Belatacept and mediastinal histoplasmosis in a kidney transplant patient

**DOI:** 10.15171/jnp.2016.15

**Published:** 2016-03-18

**Authors:** Hernán Trimarchi, Tatiana Rengel, José Andrews, Matías Paulero, Alejandro Iotti, Agustina Forastiero, Fernando Lombi, Vanesa Pomeranz, Mariano Forrester, Romina Iriarte, Iris Agorio

**Affiliations:** ^1^Nephrology Services, Hospital Británico de Buenos Aires, Buenos Aires, Argentina; ^2^Pathology Services, Hospital Británico de Buenos Aires, Buenos Aires, Argentina; ^3^Microbiology Services, Hospital Británico de Buenos Aires, Buenos Aires, Argentina

**Keywords:** Belatacept, *Histoplasma capsulatum*, Kidney transplantation

## Abstract

*Background:* In transplantation immunosuppression enhances the appearance of opportunist infections. An ideal balance between the prevention of rejection, the lowest risk of infections and the highest rates of graft survival is a continuous challenge. Lower doses of immunosuppression may diminish the risk of infections, metabolic and hemodynamic complications or even of malignancy, but may expose patients to episodes of acute rejection. New drugs are being developed to improve graft survival at the lowest risk of side effects. Belatacept has recently been introduced in kidney transplantation to inhibit the co-ligand signal of T cell stimulation. It is a drug with a safe profile, is well-tolerated and appears to improve long-term survival of kidney grafts. However, there may be an increase in opportunistic infections which may be facilitated by T cell depression, as Aspergillus sp., * Cryptococcus neoformans* or tuberculosis.

*Case Presentation:* We describe a 59-year-old female who developed fever, clinical wasting and a mediastinal mass 31 months after receiving a living non-related kidney transplant while on belatacept therapy. A mediastinal node biopsy disclosed the presence of Histoplasma capsulatum. Infection successfully resolved after appropriate antifungal treatment.

*Conclusions:* To our knowledge, this is the first reported case of *Histoplasma capsulatum* in a kidney transplanted patient on belatacept therapy

**Implication for health policy/practice/research/medical education:**

In kidney transplantation, there is an increased risk of infections. Belatacept is a novel and effective immunosuppressant with no nephrotoxic effects. We describe the first reported patient on belatacept therapy with an opportunistic infection due to *Histoplasma capsulatum*.

## 1. Introduction

Immunosuppressive drugs with specific targets are being developed in order to manipulate the immune system in a step-wise manner. In this respect, CD4 T-cells can be mainly activated by three different signals upon stimuli triggered by antigen presenting cells or cytokines, in order to amplify the immune response (1). In kidney transplantation, immunosuppressive regimes are designed to decrease the risk of acute rejection episodes by abrogating CD4 T-cell activation. In this regard, belatacept has proven to be an effective agent in the prevention of acute rejection following renal transplantation and minimizing the nephrotoxic effects on kidney function when compared to calcineurin inhibitors (2). Belatacept is a human fusion protein that combines the cytotoxic T-lymphocyte-associated antigen 4 (CTLA-4) with the constant-region fragment (Fc) of human IgG. The interaction of these surface proteins with the CD28 receptor on T-cells is necessary for T-cell activation and subsequent recognition of the presented foreign antigen. Blocking this interaction prevents antigen recognition and thereby rejection of the transplanted tissue or organ (3). While the acute rejection rates have been low albeit higher when compared to calcineurin-based protocols, the graft survival appears to be higher in the long term (4). However, reports of infections remain limited though suggestive of potentially persistent T-cell functional defects even after withdrawal of the agent. These infections to date have been limited to late onset pneumocystis carinii pneumonia (5), cytomegalovirus (6), BK virus (6), mycobacterium tuberculosis (7), cryptococcus neoformans (8) and aspergillus fumigatus (9). Herein we present a case of *Histoplasma capsulatum* infection in a living non-related kidney graft 31 months post-transplantation and discuss the potential association between histoplasmosis and belatacept.

## 2. Case Presentation

**Figure 1:**
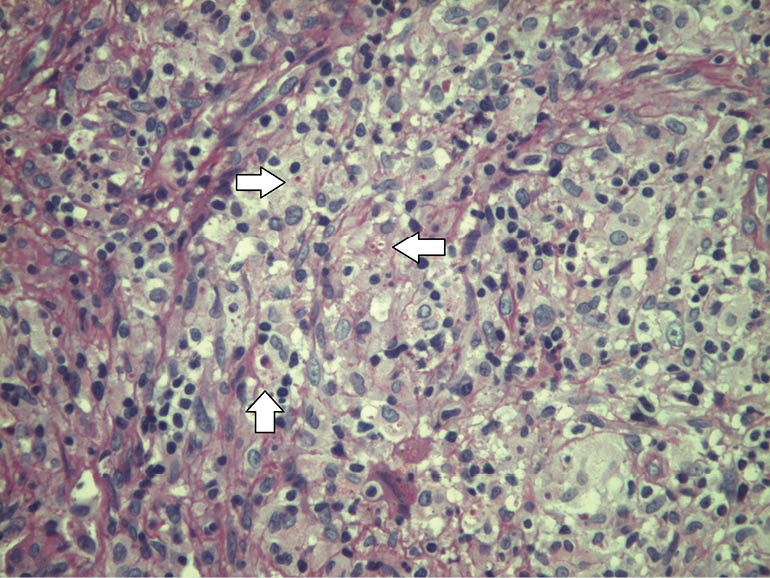
Mediastinal node biopsy. White arrows depict eosinophilic red round intracellular yeast compatible with *Histoplasma capsulatum*. PAS stain ×400.

**Figure 2:**
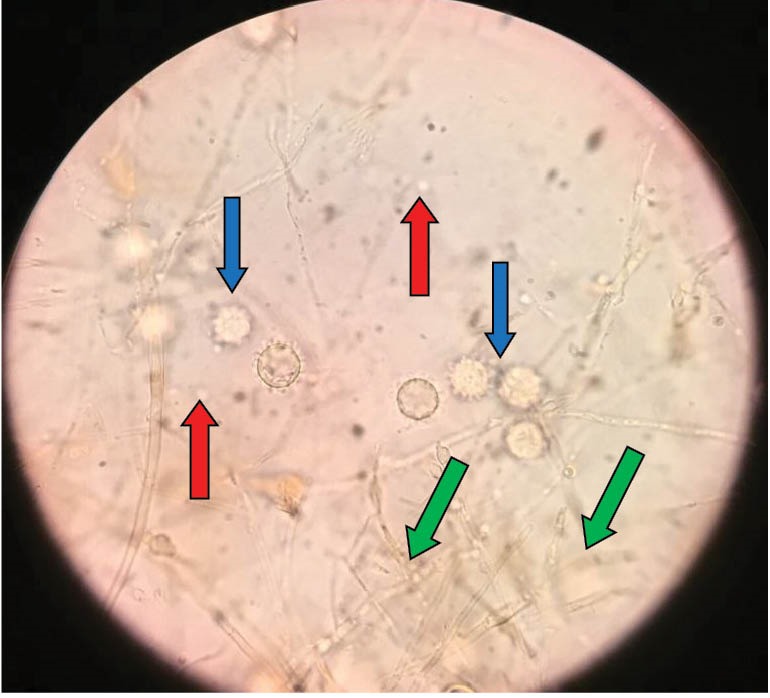
This smear displays material obtained from dissociation of colonies from the culture at 28°C of the mediastinal node. Blue arrows mark spherical tuberculated macroconidia with thick irregular outer walls (8-15 µm in diameter). Also, smooth-walled oval, small microconidia are shown in red arrows and thin branched hyaline hyphae (1.2-1.5 µm diameter) in green arrows are observed. Lactophenol ×400.

A 59-year-old female with a past history of tobacco consumption, arterial hypertension, breast cancer free of recurrence after 20 years of diagnosis and biopsy proven nephroangiosclerosis, received a kidney graft from her husband after 9 months on chronic hemodialysis. Initial immunosuppression included methylprednisolone, basiliximab, belatacept, sodium mycophenolate, while maintenance immunosuppression consisted on meprednisone 4 mg/day, belatacept 5 mg/kg/month intravenously and sodium mycophenolate 1440 mg/day without major intercurrences, except for the development of diabetes mellitus 14 months post-transplantation with insulin requirements. Thirty-one months post-transplantation she developed fever, headache episodes and weight loss. Blood and urinary cultures were negative for microbiological study. A chest x-ray disclosed a widened mediastinum. A thorax computed tomography (CT) scan disclosed enlarged mediastinal nodes. A bronchoalveolar lavage was macroscopically non-contributory, and both smears and cultures were initially negative. A mediastinoscopy was performed and a node biopsied for histological and microbiological assessment. After 24 hours small intracellular yeast compatible with *Histoplasma capsulatum* were reported in Giemsa stain in tissue samples (Figure 1). Fungal colonies were observed after 10 days of incubation of the mediastinal node. At 35°C, creamy yeast colonies were observed, while at 28°C the colonies developed aerial white cottony mycelium (Figure 2). The patient was started on intravenous amphotericin B therapy and later switched to oral fluconazol. Belatacept was not discontinued and kidney function remains normal.

## 3. Discussion

To our knowledge, this is the first reported case of *Histoplasma capsulatum* and belatacept therapy in kidney transplantation. The incidence of histoplasmosis infection in renal transplant patients ranges from <0.5% to 1.1% (10,11) as to the reported literature. In immunosuppressed patients, disseminated histoplasmosis presents a mortality between 7%-23% (12). The disease can manifest clinically due to a priming primary contact with the fungus, or as a secondary infection in subjects with a previous exposure to the fungus that it is reactivated due to a latent dormant inoculum. Exceptionally, it could be due to an undiagnosed fungal transmission present in the allograft from the donor. The clinical manifestations usually include fever, and cutaneous involvement (12). In our patient, the latter situation was excluded as the donor did not present the infection. However, we cannot discard the two first possibilities. The fact that the disease developed 31 months after transplantation may suggest it could be a primary infection, as we can speculate that a reactivation should have likely occurred in the peri-transplant period, when the immunosuppression burden was heavier.

Pulmonary involvement presents with common airway symptoms associated with diffuse lung infiltrates and/or mediastinal adenopathies (13). In this regard, bronchoalveolar lavage is mandatory, and the obtained material (lung secretions or tissue) may reveal the cultured colonies or the fungi in the biopsy. In systemic situations, the fungus can be identified in the urine (12). When all these approaches are unsuccessful, a mediastinal node must be excised and studied. In this respect, both microbiologic and histologic analysis are critical. In our patient, the diagnosis was made by both methods (Figures 1 and 2).

Belatacept has been employed to prevent acute rejection in kidney transplantation (2). Belatacept is a humanized fusion protein that combines in its molecule a surface antigen from CD4 T-cells (CTLA4) with the Fc fragment of a human IgG. The interaction between belatacept and the CD28 T-cell surface receptor is necessary for T-cell activation and the recognition of foreign antigens. The blockade of this interaction impedes the alloantigen recognition by T-cells and reduces the chances of graft rejection episodes via the co-ligand signal of CD4 T-cell activation. T-cells play a major role in the surveillance and identification of viral and fungal infections. In this regard, belatacept could have favored the fungal infection in our patient. As mentioned previously, belatacept has been associated with other fungal and mycobacterial infections (7-9). However, the reports of infections remain limited though suggestive of potentially persistent T-cell functional defects even after withdrawal of the agent (9,14-16). As to belatacept and viral infections, it has been suggested that lymphoproliferative disorders could be triggered in EBV-negative kidney receptors. A primary EBV infection in a subject on belatacept therapy could degenerate into a lymphoma. Belatacept is formally contraindicated in EBV-negative receptors (6,17).

We employed belatacept in our patient due to its advantages: Lower risk o cardiovascular disease, hypertension, it is a non-diabetogenic agent and presents lower risk of bacterial infections. The risk of early acute rejection episodes may be higher when compared to cyclosporine, but the long-term survival is higher when belatacept is employed (4).

## 4. Conclusions

In conclusion, we believe belatacept is a safe, efficient, non-nephrotoxic immunosuppressant to be employed in kidney transplantation that may present an apparently higher risk of fungal and mycobacterial infections, probably due to its mechanism of action. Despite the microbiologic background profile of the recipient is critical at the time of tailoring the immunosuppression protocol, many times infectious complications cannot be foretold.

## Authors’ contribution

Primary draft by HT, TR, JA and MP. AI and IL contributed to the histologic and microbiologic study and figures. FL, VP, MF, MF, RI and JM participated in the case discussion and assisted the patient. Manuscript edited by HT. All authors read the final version.

## Conflicts of interest

Hernán Trimarchi is a consultant to Brystol-Myers-Squibb for the product Belatacept.

## Funding/Support

No funding was obtained for the preparation of this manuscript.
